# Supplementary material for: DeepCDpred: Inter-residue distance and contact prediction for improved prediction of protein structure
Source: PLoS One. 2019 Jan 8;14(1):e0205214. doi: 10.1371/journal.pone.0205214 (PMC6324825; doi:10.1371/journal.pone.0205214)
Supplement: S1 Text — Full details of the feature vector and network architectures for DeepCDpred are explained further here, including the software used for the generation of the feature vector. Details of the structure prediction protocol and a sample from a constraint file are also given. (PDF) [file pone.0205214.s001.pdf]

Supplementary Material

DeepCDpred: Inter-residue Distance and Contact Prediction for Improved Prediction of Protein Structure.

Shuangxi Ji[1◕], Tuğçe Oruç[1◕], Liam Mead[1], Muhammad Fayyaz Rehman[1¤], Christopher Morton Thomas[1], Sam Butterworth[1,2], Peter James Winn[1*],

1 School of Biosciences, University of Birmingham, Edgbaston Birmingham, B15 2TT, UK.
2 Division of Pharmacy and Optometry, School of Health Sciences, Manchester Academic Health Sciences Centre, University of Manchester, Manchester, M13 9PL, UK.

◕These authors contributed equally to this work.
¤Current Address: Department of Chemistry, University of Sargodha, Sargodha, Pakistan.
* p.j.winn@bham.ac.uk

Generation of Sequence Alignments

We used HHblits [1, 2] to search for sequences and generate the MSAs used in this work. The the uniprot20_2016_02 sequence database was searched with E-value set to 0.001, coverage was set to 60, maximum pairwise sequence identity to 90, minimum sequence identity to 0, number of iterations to 4 and maximum number of hits allowed to pass 2nd prefilter to 500000.

For tests on MSAs with reduced Nf values the initial MSA was filtered with 80% identity threshold (by HHfilter [2]). This filtering means that the number of sequences in the MSA is the same as Meff, the effective number of sequences. For a given target Nf, Meff = Nf*$\sqrt{}$L, thus Nf*$\sqrt{}$L sequences were randomly selected from the filtered MSA, using the python function random.sample(range(2, N), Nfinal-1), where N is the initial number of sequences in the alignment, Nfinal is the desired number of sequences. As we always keep the sequence of the target protein in the reduced alignment, we pick Nfinal-1 number of sequences.

Full Details of the Feature Vector for the DeepCDpred networks.

There are 733 dimensions input into the network. For a pair of residues, (i, j), windows of length 13 amino acids are centered at i and j respectively, with another window of length 5 centred at position $(i + j)/2$. This gives $(2 * 13 + 5)$ residue positions. Each alignment position has six descriptors, three inputs representing the predicted likelihood of helix, strand and coil formation and one value of predicted solvent accessibility, which are calculated by SPIDER2 [3], the Shannon entropy and a binary value to represent whether the alignment position is part of the amino acid sequence, since as the window moves to the termini some positions in the windows will extend beyond the termini. In addition to these $(2 * 13 + 5) * (3 + 1 + 1 + 1) = 186$ features, each pair, (i, j), is also described by a statistical contact potential [4], mutual information with average product

correction (APC) [5], normalized mutual information with APC, protein chain length, the number of sequences and effective number of sequences in the MSA, the means of each of all the alpha helix, beta strand, coil, solvent accessibility scores, the frequency of each of the 20 amino acids and of gap positions in the MSA and site entropies predicted for this MSA, and the sequence separation between these two positions. Sequence separation was coded as eight binary inputs in the feature vector, representing different separation intervals. Together these provide forty entries in the feature vector. Further entries in the feature vector provide information about amino acid coupling in the broader structural context, including the scores from CCMPred [6], from QUIC (discussed in the main text), and mfDCA [7] for a window of size $13 * 13$ centered at the position pair. This sums up to $186 + 40 + (13 * 13) * 3 = 733$ features in total. The predicted HSEu from the algorithm proposed in paper [8] was tested as an alternative to rASA, but it did not help to improve contact and distance prediction.

Neural Network Architecture and Training.

For training, a nine-layer neural network architecture was used for all models (i.e. one input layer, one output layer and eight hidden layers, S1 Fig). Trainings were performed with Keras and a TensorFlow back end. In order to avoid over training, early-stopping was used. The data was randomly divided into two groups with 20% of the training feature vector used for validation. The total epochs number was set to 300 and patience was set to 40. The loss function for all networks was a binary cross-entropy function and SGD was selected as the optimizer.

Structure Prediction Protocol.

AbinitioRelax from Rosetta [9] was used for 3D structure prediction, incorporating constraints from the predicted contacts, distances and beta sheets, from our code, and the secondary structure predictions obtained from SPIDER2 [10]. Contact constraints were included for the top scoring 1.5L DeepCDpred predictions, where L is the sequence length. Three-residue and nine-residue fragments were created using Rosetta's exclude homologs function. We generated 100 candidate structures and the one with the lowest total Rosetta energy score, including constraint energy, was selected as the prediction. The Rosetta script is given elsewhere in the supplementary methods. Rosetta's standard bounded function was used as a constraint, as documented in the Rosetta manual, multiplied by a weight that depended on the DeepCDpred score, as shown in Table 1. The boundaries of the bounded function depended on the DeepCDpred score and the distance bin for which the prediction was made (Table 1), which allowed pairs with low DeepCDpred scores more easily to have values outside the distance bin boundaries. This reflects the greater tendency of pairs of residues with low DeepCDpred scores to actually deviate from the defined boundaries of the prediction bin. The formula for the upper boundary of the contact or distance constraint was determined by regressing the actual distance against the neural network score for each of these bins. The regression was performed using a subset of 435 proteins from the training set. The weights were chosen to be within the typical range of values for constraints in Rosetta [11], with predictions that were expected to be more accurate being given a higher weighting. The chosen weights were tested on a small number of proteins from the training set, and found to produce acceptable models. No optimisation of the weights was undertaken, so this represents a possible area for improvement in the future.

Since we trained neural network models for each bin separately, for some residue pairs it is possible for a given pair to score highly on more than one model. However,

only one distance or contact constraint is applied per pair, according to the rules of precedence described below. The procedure for adding distance information alongside DeepCDpred contact predictions was as follows: If the score for the 8-13 bin and/or the score for the 13-18 bin is greater than the score for the contact bin by 0.3 or more, then a distance constraint is applied representing the 8-13 or 13-18 Ångstrom distance bin, depending on which scores higher; unless the 18-23 bin range has a score that is greater than the other ranges by 0.5 or more, whereupon the residue pair has a constraint added representing the 18-23 distance range. Otherwise, a contact constraint is applied, and no distance constraint. These selection criteria reflect the differences in the observed accuracies of prediction for the different contact/distance ranges, but have not been systematically optimized.

The procedure for adding distance information alongside RaptorX contact predictions was the same as above, but using the RaptorX contact prediction score. Again, this has not been optimized and further work in this area may lead to better results. The python script which is used to prepare Rosetta constraint files is given and a short example of a constraint file is shown in the supplementary methods.

Table 1. Parameters of the contact and distance constraints.

Range /Å	DeepCDpred score (s)	Upper boundary	Lower boundary	Standard deviation	Weight
bin 0 - 8	>= 0.9	$-10.8 * s + 16.7$	3.2	0.5	2.5
	>= 0.8 & <0.9			0.7	1.5
	<0.8			1.0	1.0
bin 8 - 13	>= 0.8	$-12 * s + 23.5$	7.5	1	1.5
	<0.8			1.5	0.5
bin 13 - 18	>= 0.8	$-8.6 * s + 25.17$	$8.6 * s + 4.84$	1.5	0.8
	<0.8			1.0	0.3
bin 18 - 23	>= 0.8	$-7.2 * s + 29.2$	$7.2 * s + 11.2$	1.5	0.6
	<0.8			1.0	0.3

For structure predictions using MetaPSICOV, RaptorX and NeBcon, the top 1.5L contact predictions were chosen, which allows us to make comparisons with DeepCDpred.

Generation of non-topologous test set

We aimed to have the largest independent topology set that we could create. Since we were comparing across RaptorX, MetaPSICOV and DeepCDpred, the set should be topologously independent of all of their training sets. As outlined below, due to the size of the RaptorX training set this left few possibilities. There are 1391 topology classes in the CATH (v4.0.0) database and 1064 of them were used in RaptorX, MetaPSICOV, DeepCDpred training and validation sets. 12234 protein chains were detected belonging to remaining 327 topology classes. Then, we used the PISCES server to cull the proteins with 25% or less sequence identity, X-ray resolution lower than 2.5 Å and chains with length shorter than 400 amino acids and longer than 40 amino acids. This left us with 108 proteins. Among them, we removed the ones with missing residues or atoms in the structure. That resulted 50 proteins to be used as additional test set. For the expanded non-topologous set, we added proteins topologous to proteins in the MetaPSICOV training set, but not the DeepCDpred or RaptorX training sets. Applying the same filters as above allowed us to add 11 more proteins to the non topologous set (PDB IDs are given in Table 5).

Table 2. Comparison of average TM-scores of the structure pools with 100 vs. 200 models.

	Lowest Rosetta Energy[1]			Model with highest TM compared to experimental structure		
	Average TM from 100	Average TM from 200	p-value[2]	Average TM from 100	Average TM from 200	p-value[2]
RaptorX contact only (108)	0.667	0.665	0.320	0.766	0.775	0.04
RaptorX + Distance (108)	0.720	0.737	0.008	0.780	0.786	$7\text{x}10^{-5}$
RaptorX contact only (50)	0.493	0.494	0.831	0.557	0.568	$1\text{x}10^{-5}$
RaptorX + Distance (50)	0.516	0.515	0.850	0.561	0.576	$8\text{x}10^{-5}$

[1]Average TM, compared to experimental structure, of the model with lowest Rosetta energy model for each of the 108 proteins and the 50 proteins in the test sets, when using a pool of 100 or 200 models, per protein, to select from. [2]Paired t-test.

Software Used

The following lists the software, as well as related settings, that were used for residue contact/distance prediction and 3D structure prediction.

MI_APC. Mutual Information with the average product correlation (MI_APC), as described by Dunn *et al.* [12], was calculated using a script in the MetaPSICOV source code, which was downloaded from http://bioinfadmin.cs.ucl.ac.uk/downloads/MetaPSICOV/.

mfDCA. mfDCA calculations were generated using the implementation provided by the FreeContact package (downloaded from ftp://rostlab.org/free//freecontact-1.0.21.tar.xz) with default parameters.

QUIC. The source code of QUIC was downloaded from http://www.cs.utexas.edu/~sustik/QUIC/QUIC_MEX_1.1.tar and consisted of a mixture of MATLAB and C scripts. It was rewritten solely in C in order to speed up the calculation. OpenMP was also adopted to allow it to run in parallel. Parameters in the code such as the regularization parameter and the parameter of tolerance were kept the same as in the downloaded code, the tolerance being 0.004 and the regularization being L1, with regularisation parameter 0.2

CCMpred. CCMpred was downloaded from https://github.com/soedinglab/CCMpred and run with default parameters.

Rosetta. The source code of version 3.7 was downloaded from https://rosettacommons.org and compiled into executable files using the openMPI library.

SPIDER2. The source code of SPIDER2 and the training dataset of SPIDER2 were downloaded from http://sparks-lab.org/server/SPIDER2/. Protein secondary structure predictions were generated using default settings.

Blastpgp. When using SPIDER2 to predict the secondary structure of the query protein, Blastpgp, which comes from the BLAST family, was required to calculate the

PSSM, which was fed into the neural network model in SPIDER2. Blastpgp version 2.2.26 was used here and uniref90 (November 2016) was used as the sequence database.

HHblits. HHsuite (version 2.0.16) was downloaded from the github (https://github.com/soedinglab/hh-suite). HHblits requires a program-specific protein sequence database of pre-calculated HMM profiles, with each HMM relating to a sequence cluster from the UniProt sequence database. Here, the version released in February 2016 (filename: uniprot20_2016_02) was used. The parameter settings used in HHblits were: iteration: 4, E-value: 0.001, minimum coverage with the query sequence: 60%, maximum pairwise sequence identity: 90%.

Table 3. PDB ID list of the test set with 108 proteins.

1a3aA	1cc8A	1dsxA	1gzcA	1im5A	1ku3A	1p90A	1vjkA
1aapA	1chdA	1eazA	1h2eA	1j3aA	1kw4A	1pchA	1vmbA
1abaA	1cjwA	1ej8A	1h4xA	1jfuA	1lm4A	1qf9A	1vp6A
1ag6A	1ckeA	1f6bA	1hdoA	1jl1A	1lo7A	1qjpA	1w0hA
1aoeA	1ctfA	1fcyA	1hfcA	1jo0A	1m4jA	1r26A	1whiA
1atzA	1cxyA	1fk5A	1hh8A	1jo8A	1m8aA	1roaA	1wjxA
1avsA	1cznA	1fl0A	1htwA	1josA	1mk0A	1rw1A	1wkcA
1bdoA	1d0qA	1fvgA	1hxnA	1jwqA	1mugA	1smxA	1xffA
1bebA	1d1qA	1fx2A	1i1jA	1jyhA	1nb9A	1svyA	2cuaA
1behA	1d4oA	1g2rA	1i1nA	1k6kA	1ne2A	1t8kA	2phyA
1bkrA	1dixA	1g9oA	1i4jA	1k7jA	1npsA	1tifA	1c44A
1dlwA	1gmiA	1i58A	1kq6A	1nrvA	1tqgA	1c52A	1dmgA
1gmxA	1i71A	1kqrA	1ny1A	1tqhA	1c9oA	1dqgA	1gz2A
1iibA	1ktgA	1o1zA	1vfyA				

Table 4. PDB ID list of the test set with 50 proteins.

1b12A	1ckmA	1d0qA	1dd9A	1dmgA
1e1hA	1e1hB	1g2rA	1hufA	1i71A
1inpA	1io1A	1j3aA	1o9iA	1okcA
1r7lA	1rajA	1sknP	1svbA	1tgrA
1w2yA	1whiA	1wjxA	1yrtA	1yu5X
1ywmA	2j7aC	2p84A	2rhkC	2vnlA
2wqiA	3bl9B	3bqwA	3girA	3hrdB
3o79A	3pn3A	3rioA	3rlfG	3ts2A
3vtoQ	3x02A	3x34A	4x8yA	4xb4A
4ymuC	4z6mA	5b66O	5hobA	5hocA

Table 5. PDB ID list of additional test set with 11 proteins.

1af7A	1ddgA	1dl5A	1fjrA	1fn9A
1h3iA	1h6wA	1iomA	1noyA	1rkuA
1rzhA				

Network Training Script

The python script for training the neural network is given in the file train_network.py.

Rosetta Scripts

Parameter settings in the Rosetta protocol file: The Rosetta script that is used to generate structures via Abinitio relax is given in the file flags_for_rosetta_ab_initio.

Example of a constraint file:

AtomPair CB 77 CB 85 SCALARWEIGHTEDFUNC 0.50 BOUNDED 7.50 15.01 1.5 NOE

AtomPair CB 21 CB 47 SCALARWEIGHTEDFUNC 1.00 BOUNDED 3.20 10.07 1.0 NOE

AtomPair CB 25 CB 31 SCALARWEIGHTEDFUNC 2.50 BOUNDED 3.20 6.12 0.5 NOE

AtomPair CB 22 CB 44 SCALARWEIGHTEDFUNC 2.50 BOUNDED 3.20 6.22 0.5 NOE

AtomPair CB 24 CB 44 SCALARWEIGHTEDFUNC 1.00 BOUNDED 3.20 8.77 1.0 NOE

AtomPair CB 11 CB 56 SCALARWEIGHTEDFUNC 0.50 BOUNDED 7.50 14.44 1.5 NOE

AtomPair CB 1 CB 17 SCALARWEIGHTEDFUNC 0.50 BOUNDED 7.50 15.61 1.5 NOE

AtomPair CB 48 CB 86 SCALARWEIGHTEDFUNC 1.00 BOUNDED 3.20 8.60 1.0 NOE

AtomPair CB 25 CB 65 SCALARWEIGHTEDFUNC 0.50 BOUNDED 7.50 15.80 1.5 NOE

AtomPair CB 10 CB 46 SCALARWEIGHTEDFUNC 0.50 BOUNDED 7.50 15.36 1.5 NOE

AtomPair CB 9 CB 62 SCALARWEIGHTEDFUNC 1.00 BOUNDED 3.20 9.61 1.0 NOE

AtomPair CB 24 CB 66 SCALARWEIGHTEDFUNC 0.50 BOUNDED 7.50 14.45 1.5 NOE

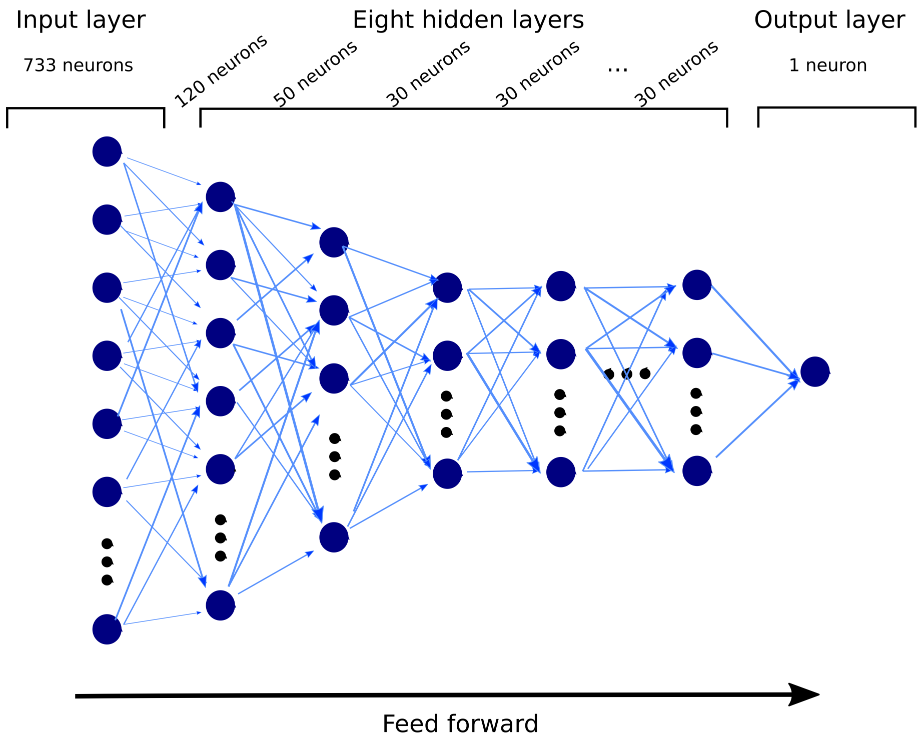

Fig 1. The architecture of the neural network model adopted for amino acid contact and distance predictions in this study.

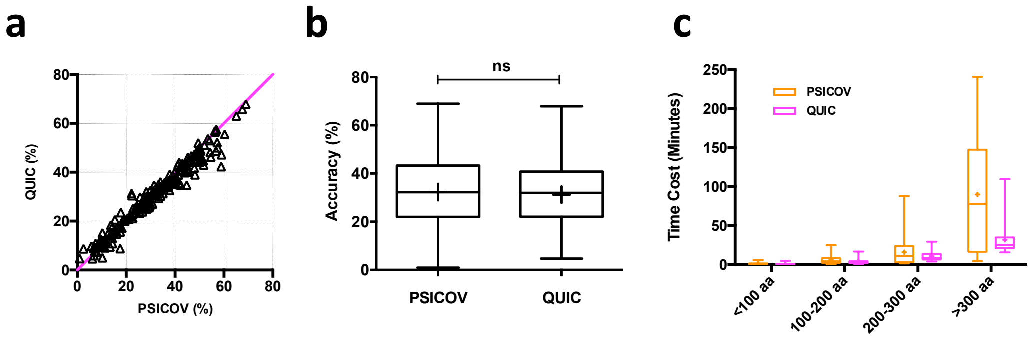

Fig 2. Contact prediction accuracy and speed comparisons between PSICOV and QUIC. 221 proteins from the training set were chosen for the comparisons and the accuracies of the top 1.5L amino acid contact predictions of each protein for both PSICOV and QUIC is shown in graph (a). Graph (b) shows the average contact prediction accuracies of the top scoring 1.5L amino acid pairs. (a) and (b) indicate there is little difference between PSICOV and QUIC for amino acid contact prediction. (c), based on the same computer (8-core i7-3770, 32 GB RAM), PSICOV took 16.9 minutes to complete the contact prediction for each protein on average; while QUIC only took 6.9 minutes; especially for large proteins (>300 amino acids), QUIC is much faster than PSICOV.

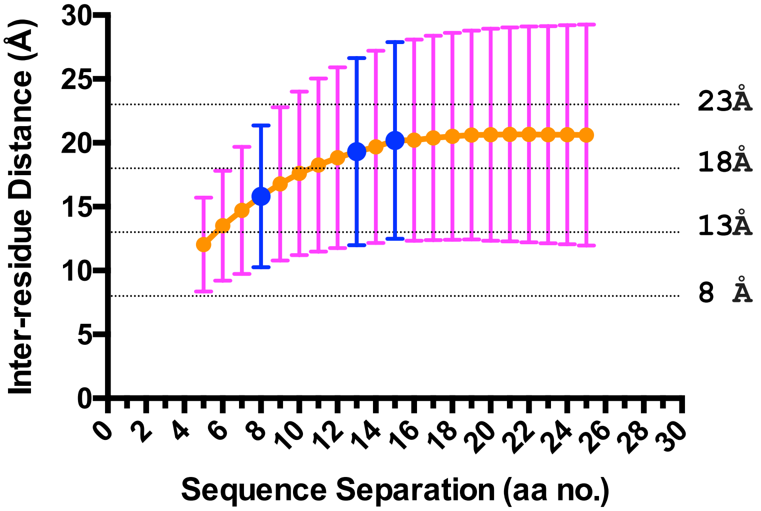

Fig 3. The distribution of inter-residue distance with respect to the sequence separation of a pair of residues. The mean and standard deviation for 435 experimental protein structures from the training set are shown. The three blue highlighted sequence separations (8, 13 and 15) are the minimum sequence separation cut-offs chosen for distance predictions in bin 8-13, 13-18 and 18-23, respectively.

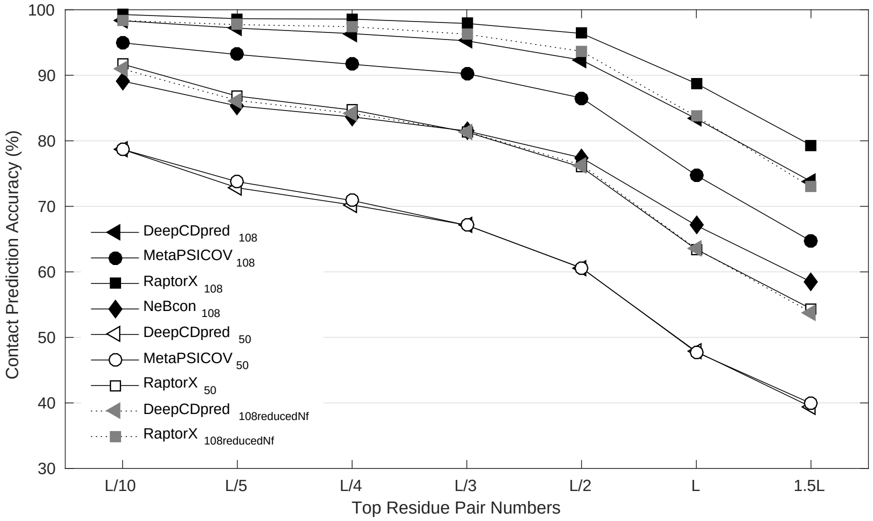

Fig 4. Contact prediction accuracies of both test sets (with 108 and 50 proteins). The average accuracies for the test set with 108 proteins is higher than the test set with 50 proteins. The 108 protein test set had the number of sequences in each MSA reduced to give an average Nf value similar to that of the MSAs for the 50 protein test set. Reducing the Nf value decreased the prediction accuracy of DeepCDpred and RaptorX, however the drop in accuracy of the former was much larger than that of the latter.

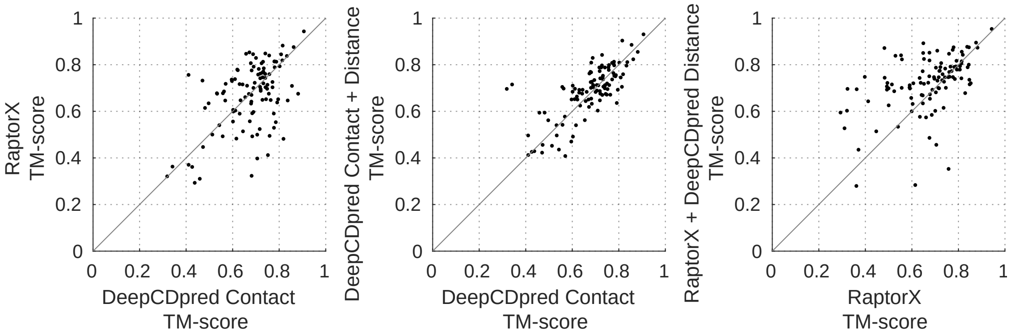

Fig 5. Addition of distance constraints improves the model quality of both DeepCDpred and RaptorX when the model is selected with Rosetta energy score. The calculations are for the test set of 108 proteins. The graphs show comparison of the TM-score with respect to experimental structures of lowest energy models predicted using constraints from RaptorX, DeepCDpred contact only, DeepCDpred contact + distance and RaptorX contact + DeepCDpred distance predictions. For each test protein 100 structures were generated by Rosetta.

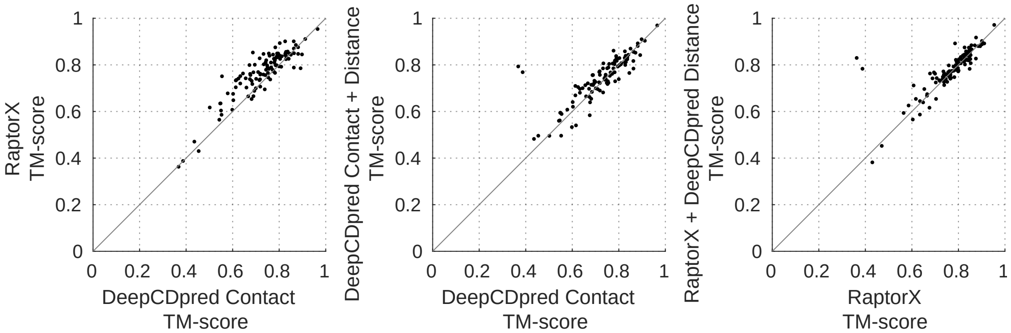

Fig 6. Addition of distance constraints improves the model quality of both DeepCDpred and RaptorX when the model with highest TM-score is selected. The calculations are for the test set of 108 proteins. The graphs show comparison of the TM-score with respect to experimental structures of the best models predicted using constraints from RaptorX, DeepCDpred contact only, DeepCDpred contact + distance and RaptorX contact + DeepCDpred distance predictions. For each test protein 100 structures were generated by Rosetta.

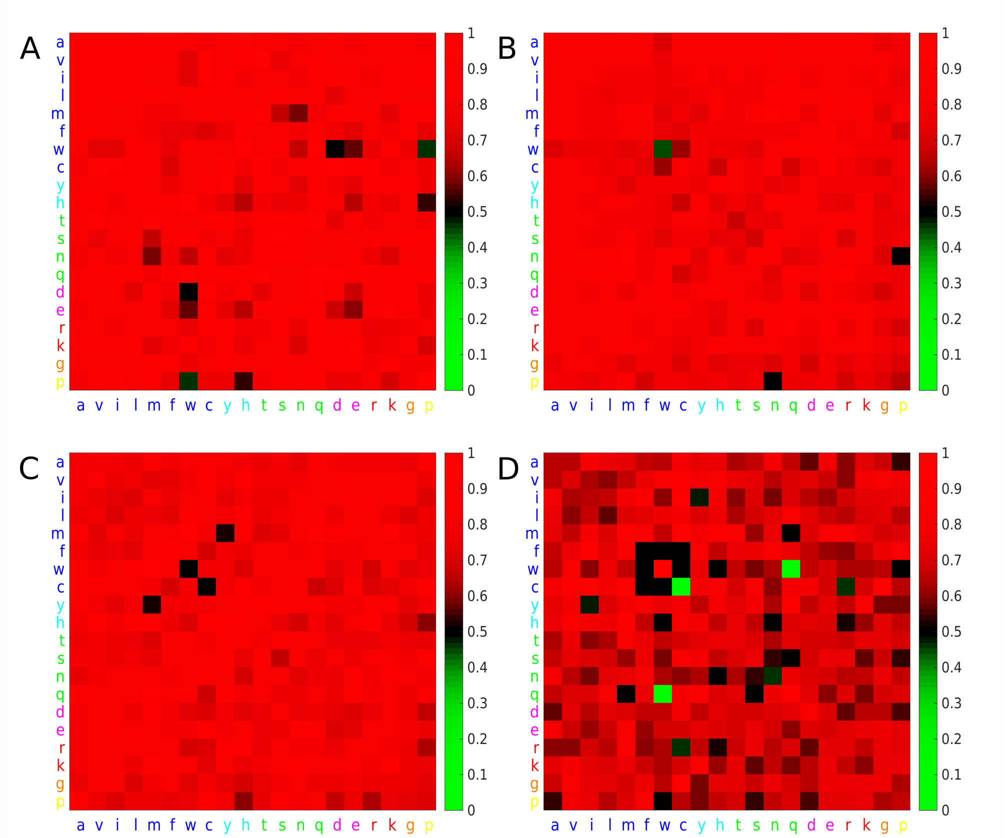

Fig 7. The precision of predicting contacts and distances between different residue types for (a) 0-8 Å, (b) 8-13 Å, (c) 13-18 Å, (d) 18-23 Å. The scale is given on the right hand side for each plot. Precision is calculated as the number of correctly predicted contacts for that pair of amino acid types divided by the total number of contact predictions for that pair for the predictions with >=0.7 network score.

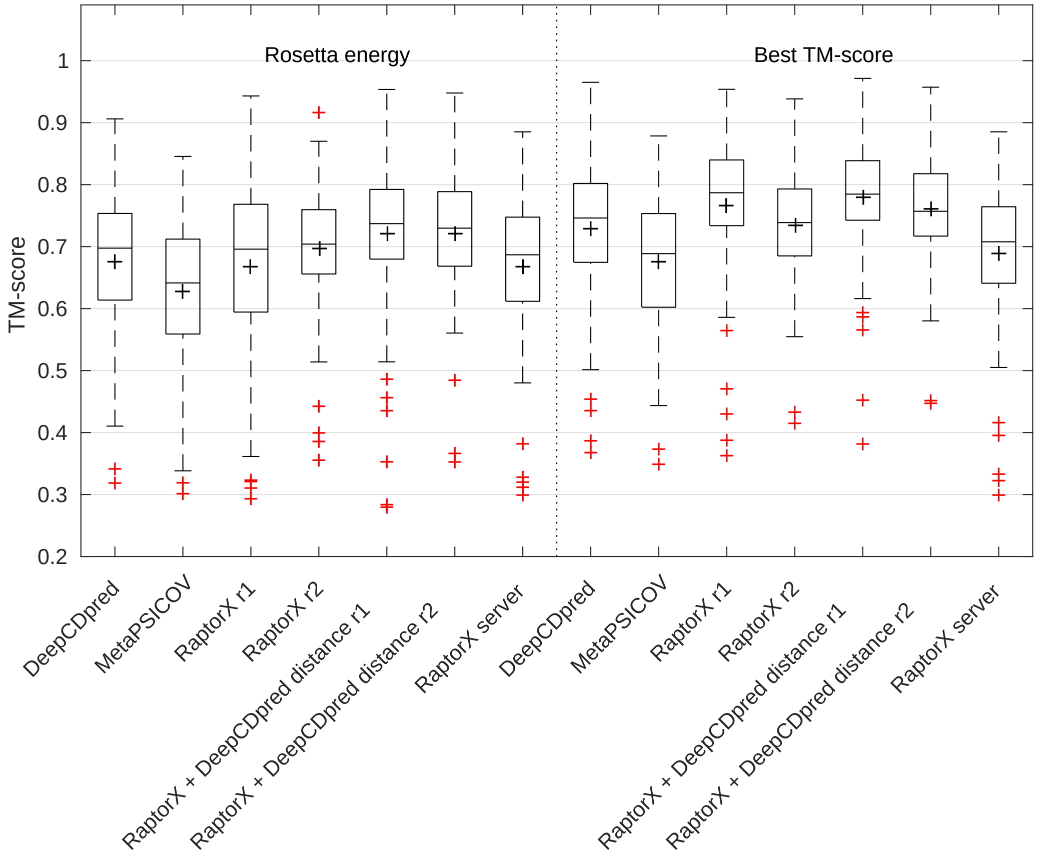

Fig 8. TM-scores of the models generated with different tools. Structure predictions for Rosetta contact and Rosetta contact plus DeepCDpred distances were replicated (replica1 (r1) and replica2 (r2)). For Rosetta server predictions models were selected either by the lowest energy score (CNS score) or the best model among the 5 structures that the server provides. For all other prediction methods, models were selected either with the lowest Rosetta energy or the best TM-score. The calculations were performed for the test set of 108 proteins. The upper and the lower edges of the boxes indicate the 25th and 75th percentiles, respectively. The medians are shown with the central lines, the means are shown with black '+' signs and the outliers are shown with red '+' signs. Even though the first set of best models which were generated with the restraints of RaptorX contact predictions (RaptorX r1) are significantly better than the best models generated with DeepCDpred contact predictions, replication of the structure predictions with RaptorX contacts (RaptorX r2) resulted in no significantly different average TM-score than the predictions performed with DeepCDpred contacts (paired t-test p-value: 0.507). The results from the RaptorX server were on average worse than all other calculations except the use of MetaPSICOV contact restraints together with Rosetta, presumably because CNS, used by the RaptorX server, is not as good at modelling structures as Rosetta is.

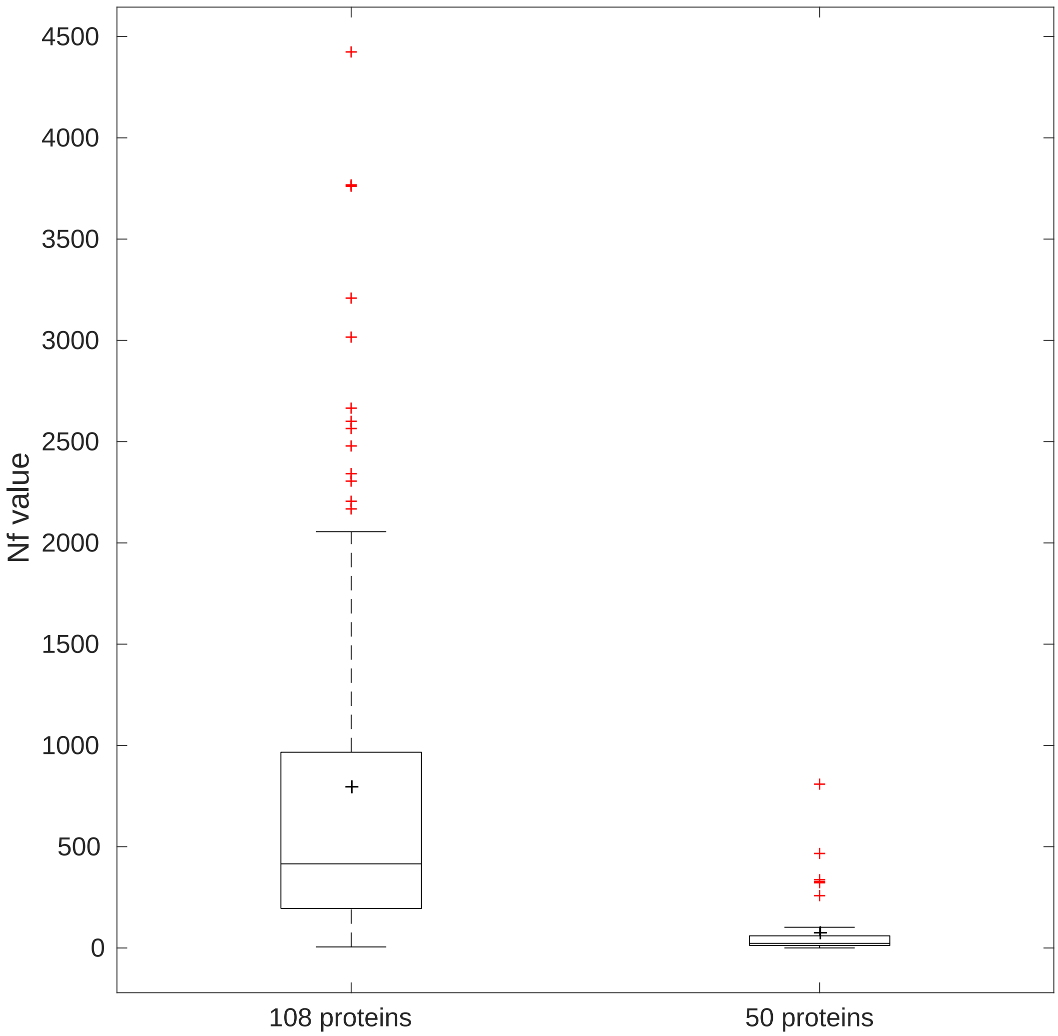

Fig 9. Nf value distributions of both test sets (with 108 and 50 proteins). The upper and the lower edges of the boxes indicate the 25th and 75th percentiles, respectively. The medians are shown with the central lines, the means are shown with black '+' signs and the outliers are shown with red '+' signs.

References

1. Remmert M, Biegert A, Hauser A, Söding J. HHblits: lightning-fast iterative protein sequence searching by HMM-HMM alignment. Nature Methods. 2011;9:173 EP –.

2. Zimmermann L, Stephens A, Nam SZ, Rau D, Kübler J, Lozajic M, et al. A Completely Reimplemented MPI Bioinformatics Toolkit with a New HHpred Server at its Core. Journal of Molecular Biology. 2018;430(15):2237 – 2243. doi:https://doi.org/10.1016/j.jmb.2017.12.007.

3. Yang Y, Heffernan R, Paliwal K, Lyons J, Dehzangi A, Sharma A, et al. In: Zhou Y, Kloczkowski A, Faraggi E, Yang Y, editors. SPIDER2: A Package to Predict Secondary Structure, Accessible Surface Area, and Main-Chain Torsional Angles by Deep Neural Networks. New York, NY: Springer New York; 2017. p. 55–63. Available from: https://doi.org/10.1007/978-1-4939-6406-2_6.

4. Betancourt MR, Thirumalai D. Pair potentials for protein folding: Choice of reference states and sensitivity of predicted native states to variations in the interaction schemes. Protein Science. 2008;8(2).

5. Buslje CM, Santos J, Delfino JM, Nielsen M. Correction for phylogeny, small number of observations and data redundancy improves the identification of coevolving amino acid pairs using mutual information. Bioinformatics. 2009;25(9):1125–1131. doi:10.1093/bioinformatics/btp135.

6. Seemayer S, Gruber M, Söding J. CCMpred—fast and precise prediction of protein residue–residue contacts from correlated mutations. Bioinformatics. 2014;30(21):3128–3130. doi:10.1093/bioinformatics/btu500.

7. Morcos F, Pagnani A, Lunt B, Bertolino A, Marks DS, Sander C, et al. Direct-coupling analysis of residue coevolution captures native contacts across many protein families. Proceedings of the National Academy of Sciences. 2011;108(49):E1293–E1301. doi:10.1073/pnas.1111471108.

8. Heffernan R, Dehzangi A, Lyons J, Paliwal K, Sharma A, Wang J, et al. Highly accurate sequence-based prediction of half-sphere exposures of amino acid residues in proteins. Bioinformatics. 2016;32(6):843–849. doi:10.1093/bioinformatics/btv665.

9. Simons KT, Bonneau R, Ruczinski I, Baker D. Ab initio protein structure prediction of CASP III targets using ROSETTA. Proteins: Structure, Function, and Bioinformatics. 1999;37(3):171–176.

10. Heffernan R, Paliwal K, Lyons J, Dehzangi A, Sharma A, Wang J, et al. Improving prediction of secondary structure, local backbone angles, and solvent accessible surface area of proteins by iterative deep learning. Scientific Reports. 2015;5:11476 EP –.

11. Ovchinnikov S, Kinch L, Park H, Liao Y, Pei J, Kim DE, et al. Large-scale determination of previously unsolved protein structures using evolutionary information. eLife. 2015;4:e09248. doi:10.7554/eLife.09248.

12. Dunn SD, Wahl LM, Gloor GB. Mutual information without the influence of phylogeny or entropy dramatically improves residue contact prediction. Bioinformatics. 2008;24(3):333–340. doi:10.1093/bioinformatics/btm604.
