# Supplementary material for: DeepCDpred: Inter-residue distance and contact prediction for improved prediction of protein structure
Source: PLoS One. 2019 Jan 8;14(1):e0205214. doi: 10.1371/journal.pone.0205214 (PMC6324825; doi:10.1371/journal.pone.0205214)
Supplement: S1 Table — (PDF) [file pone.0205214.s002.pdf]

Table 1. Parameters of the contact and distance constraints.

Range /Å	DeepCDpred score (s)	Upper boundary	Lower boundary	Standard deviation	Weight
	>= 0.9			0.5	2.5
bin 0 - 8	>= 0.8 & <0.9	$-10.8 * s + 16.7$	3.2	0.7	1.5
	<0.8			1.0	1.0
bin 8 - 13	>= 0.8	$-12 * s + 23.5$	7.5	1	1.5
	<0.8			1.5	0.5
bin 13 - 18	>= 0.8	$-8.6 * s + 25.17$	$8.6 * s + 4.84$	1.5	0.8
	<0.8			1.0	0.3
bin 18 - 23	>= 0.8	$-7.2 * s + 29.2$	$7.2 * s + 11.2$	1.5	0.6
	<0.8			1.0	0.3
