# Supplementary material for: DeepCDpred: Inter-residue distance and contact prediction for improved prediction of protein structure
Source: PLoS One. 2019 Jan 8;14(1):e0205214. doi: 10.1371/journal.pone.0205214 (PMC6324825; doi:10.1371/journal.pone.0205214)
Supplement: S2 Table — (PDF) [file pone.0205214.s003.pdf]

Table 2. Comparison of average TM-scores of the structure pools with 100 vs. 200 models.

	Lowest Rosetta Energy[1]			Model with highest TM compared to experimental structure		
	Average TM from 100	Average TM from 200	p-value[2]	Average TM from 100	Average TM from 200	p-value[2]
RaptorX contact only (108)	0.667	0.665	0.320	0.766	0.775	0.04
RaptorX + Distance (108)	0.720	0.737	0.008	0.780	0.786	$7x10^{-5}$
RaptorX contact only (50)	0.493	0.494	0.831	0.557	0.568	$1x10^{-5}$
RaptorX + Distance (50)	0.516	0.515	0.850	0.561	0.576	$8x10^{-5}$

[1]Average TM, compared to experimental structure, of the model with lowest Rosetta energy model for each of the 108 proteins and the 50 proteins in the test sets, when using a pool of 100 or 200 models, per protein, to select from. [2]Paired t-test.
