# Supplementary material for: DeepCDpred: Inter-residue distance and contact prediction for improved prediction of protein structure
Source: PLoS One. 2019 Jan 8;14(1):e0205214. doi: 10.1371/journal.pone.0205214 (PMC6324825; doi:10.1371/journal.pone.0205214)
Supplement: S3 Table — (PDF) [file pone.0205214.s004.pdf]

Table 3. PDB ID list of the test set with 108 proteins.

1a3aA	1cc8A	1dsxA	1gzcA	1im5A	1ku3A	1p90A	1vjkA
1aapA	1chdA	1eazA	1h2eA	1j3aA	1kw4A	1pchA	1vmbA
1abaA	1cjwA	1ej8A	1h4xA	1jfuA	1lm4A	1qf9A	1vp6A
1ag6A	1ckeA	1f6bA	1hdoA	1jl1A	1lo7A	1qjpA	1w0hA
1aoeA	1ctfA	1fcyA	1hfcA	1jo0A	1m4jA	1r26A	1whiA
1atzA	1cxyA	1fk5A	1hh8A	1jo8A	1m8aA	1roaA	1wjxA
1avsA	1cznA	1fl0A	1htwA	1josA	1mk0A	1rw1A	1wkcA
1bdoA	1d0qA	1fvgA	1hxnA	1jwqA	1mugA	1smxA	1xffA
1bebA	1d1qA	1fx2A	1i1jA	1jyhA	1nb9A	1svyA	2cuaA
1behA	1d4oA	1g2rA	1i1nA	1k6kA	1ne2A	1t8kA	2phyA
1bkrA	1dixA	1g9oA	1i4jA	1k7jA	1npsA	1tifA	1c44A
1dlwA	1gmiA	1i58A	1kq6A	1nrvA	1tqgA	1c52A	1dmgA
1gmxA	1i71A	1kqrA	1ny1A	1tqhA	1c9oA	1dqgA	1gz2A
1iibA	1ktgA	1o1zA	1vfyA				
