# Supplementary material for: DeepCDpred: Inter-residue distance and contact prediction for improved prediction of protein structure
Source: PLoS One. 2019 Jan 8;14(1):e0205214. doi: 10.1371/journal.pone.0205214 (PMC6324825; doi:10.1371/journal.pone.0205214)
Supplement: S4 Table — (PDF) [file pone.0205214.s005.pdf]

Table 4. PDB ID list of the test set with 50 proteins.

1b12A	1ckmA	1d0qA	1dd9A	1dmgA
1e1hA	1e1hB	1g2rA	1hufA	1i71A
1inpA	1io1A	1j3aA	1o9iA	1okcA
1r7lA	1rajA	1sknP	1svbA	1tgrA
1w2yA	1whiA	1wjxA	1yrtA	1yu5X
1ywmA	2j7aC	2p84A	2rhkC	2vnlA
2wqiA	3bl9B	3bqwA	3girA	3hrdB
3o79A	3pn3A	3rioA	3rlfG	3ts2A
3vtoQ	3x02A	3x34A	4x8yA	4xb4A
4ymuC	4z6mA	5b66O	5hobA	5hocA
