# Supplementary material for: DeepCDpred: Inter-residue distance and contact prediction for improved prediction of protein structure
Source: PLoS One. 2019 Jan 8;14(1):e0205214. doi: 10.1371/journal.pone.0205214 (PMC6324825; doi:10.1371/journal.pone.0205214)
Supplement: S5 Table — (PDF) [file pone.0205214.s006.pdf]

Table 5. PDB ID list of additional test set with 11 proteins.

1af7A	1ddgA	1dl5A	1fjrA	1fn9A
1h3iA	1h6wA	1iomA	1noyA	1rkuA
1rzhA				
