# Supplementary figures and images for: DeepCDpred: Inter-residue distance and contact prediction for improved prediction of protein structure
Source: PLoS One. 2019 Jan 8;14(1):e0205214. doi: 10.1371/journal.pone.0205214 (PMC6324825; doi:10.1371/journal.pone.0205214)

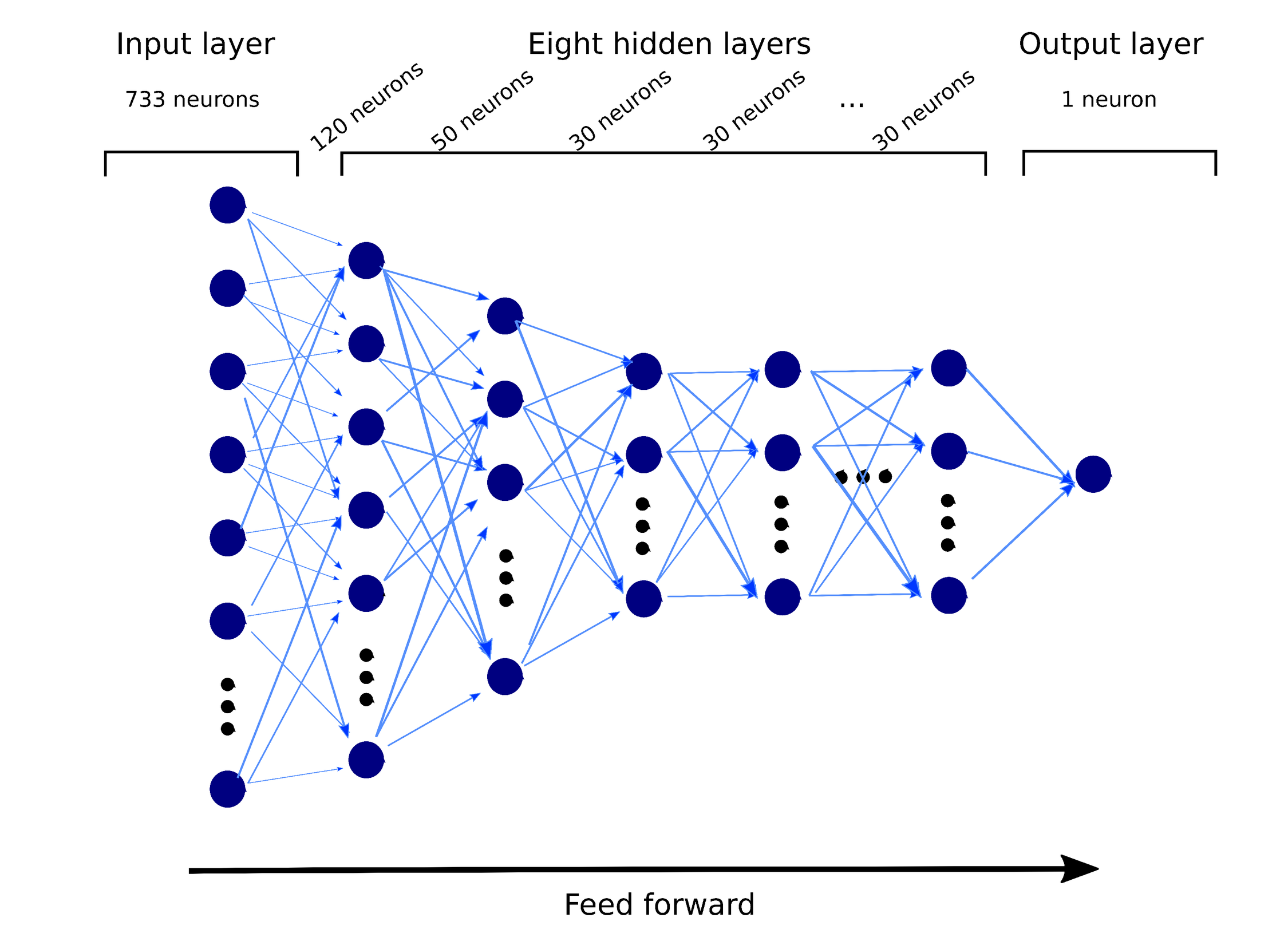

Supplement: S1 Fig — (TIF) [file pone.0205214.s007.tif]

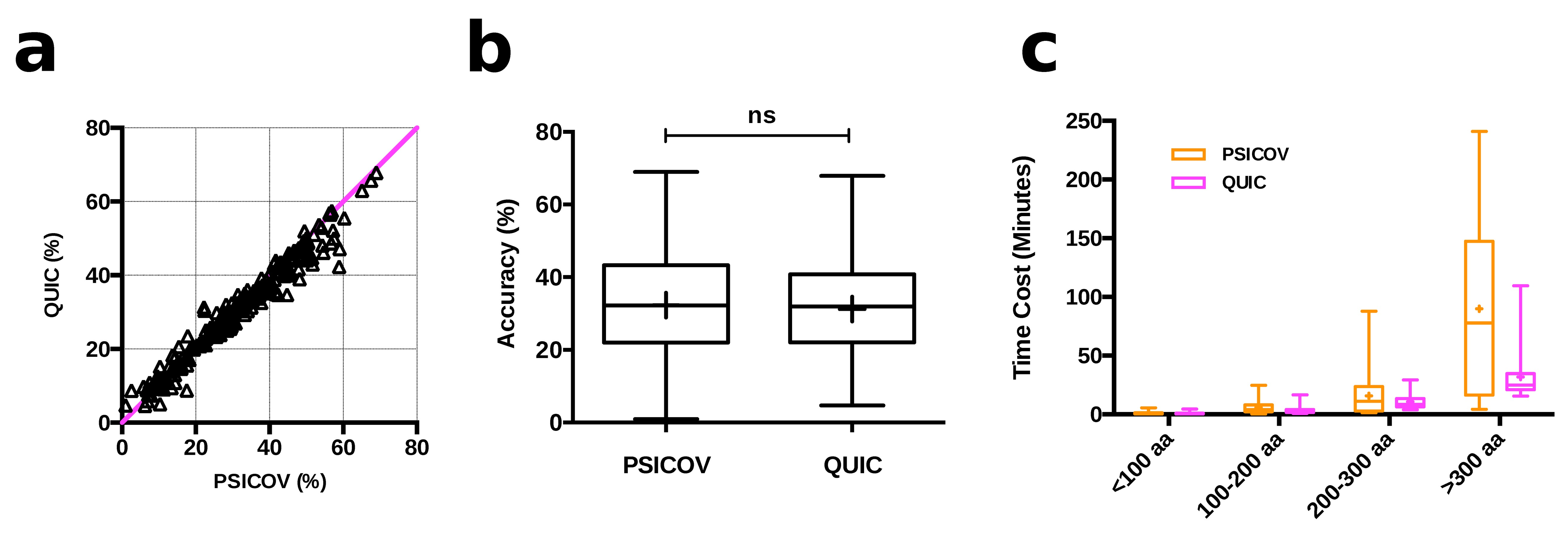

Supplement: S2 Fig — 221 proteins from the training set were chosen for the comparisons and the accuracies of the top 1.5L amino acid contact predictions of each protein for both PSICOV and QUIC is shown in graph (a). Graph (b) shows the average contact prediction accuracies of the top scoring 1.5L amino acid pairs. (a) and (b) indicate there is little difference between PSICOV and QUIC for amino acid contact prediction. (c), based on the same computer (8-core i7-3770, 32 GB RAM), PSICOV took 16.9 minutes to complete the contact prediction for each protein, on average; while QUIC only took 6.9 minutes; especially for large proteins (>300 amino acids), QUIC is much faster than PSICOV. (TIF) [file pone.0205214.s008.tif]

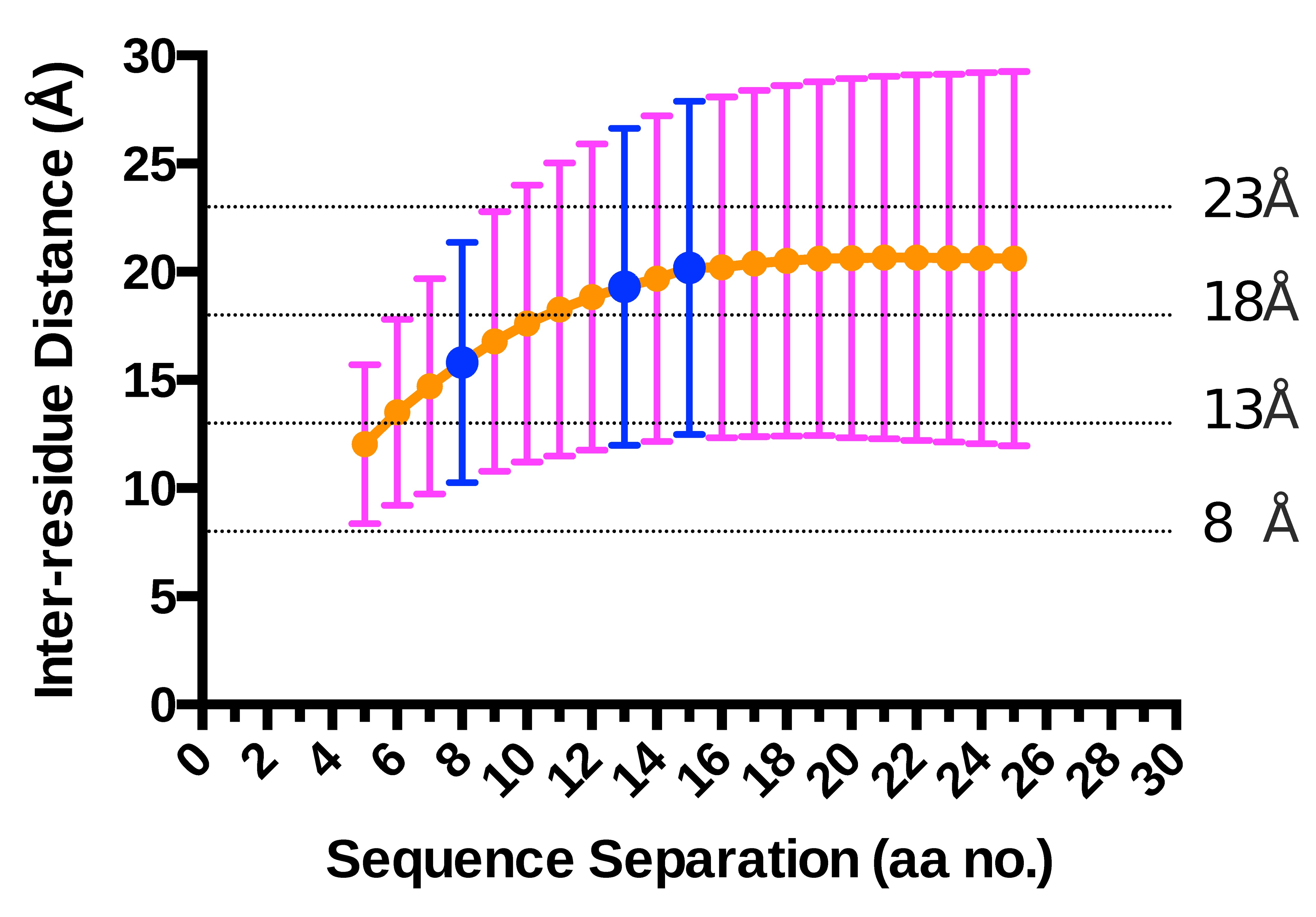

Supplement: S3 Fig — The mean and standard deviation for 435 experimental protein structures from the training set are shown. The three blue highlighted sequence separations (8, 13 and 15) are the minimum sequence separation cut-offs chosen for distance predictions in bin 8-13, 13-18 and 18-23, respectively. (TIF) [file pone.0205214.s009.tif]

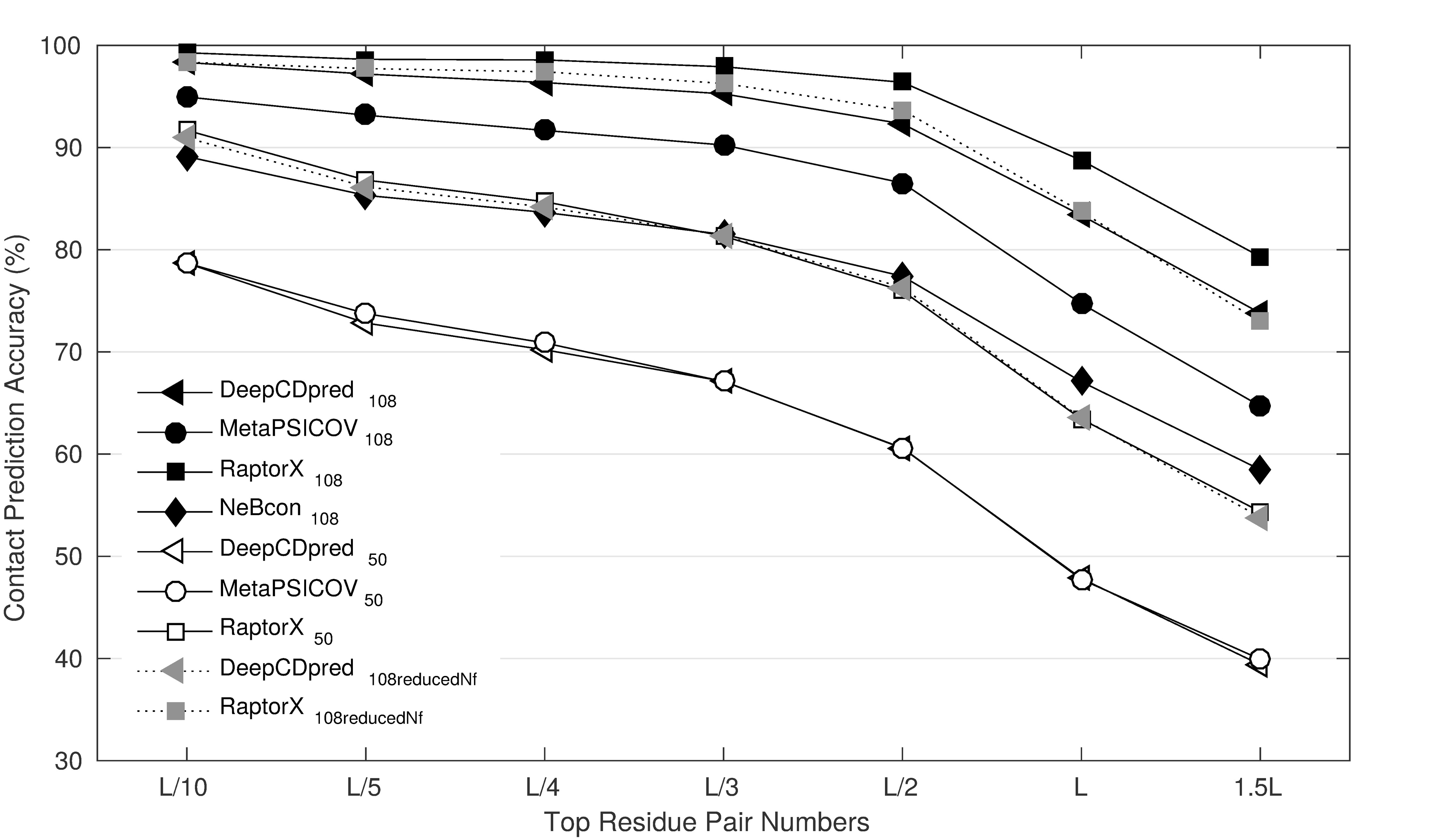

Supplement: S4 Fig — The average accuracies for the test set with 108 proteins is higher than the test set with 50 proteins. The 108 protein test set had the number of sequences in each MSA reduced to give an average Nf value similar to that of the MSAs for the 50 protein test set. Reducing the Nf value decreased the prediction accuracy of DeepCDpred and RaptorX, however the drop in accuracy of the former was much larger than that of the latter. (TIF) [file pone.0205214.s010.tif]

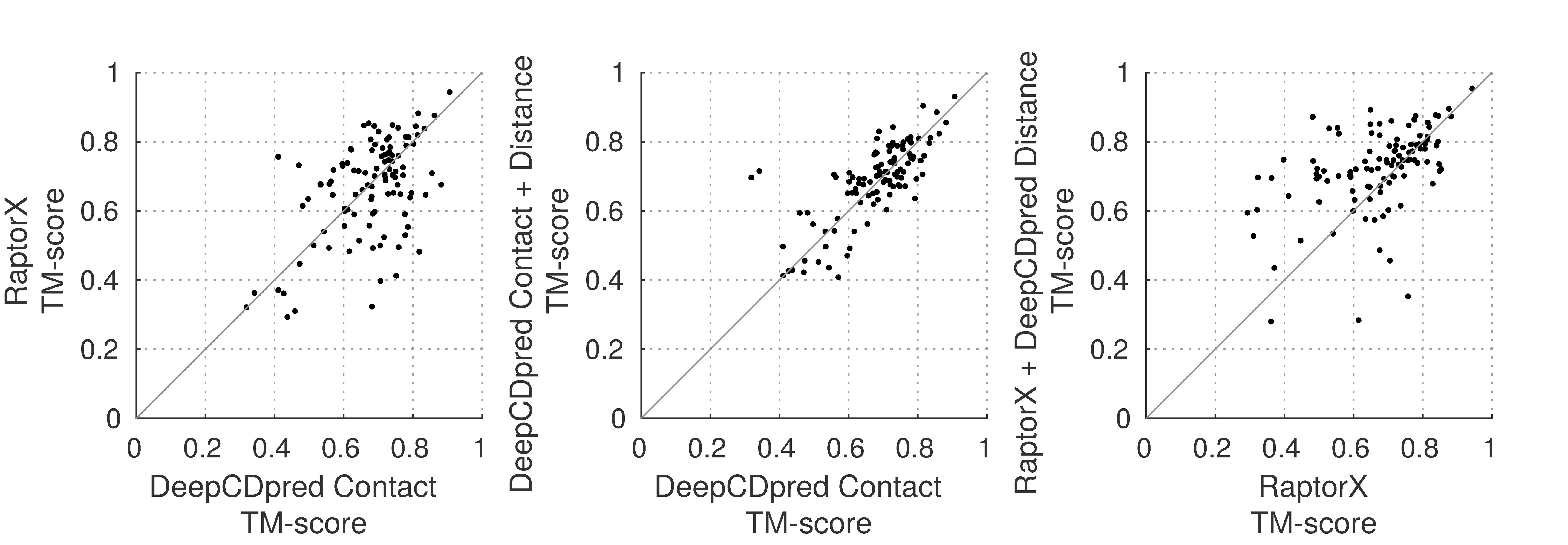

Supplement: S5 Fig — The calculations are for the test set of 108 proteins. The graphs show comparison of the TM-score with respect to experimental structures of lowest energy models predicted using constraints from RaptorX, DeepCDpred contact only, DeepCDpred contact + distance and RaptorX contact + DeepCDpred distance predictions. For each test protein 100 structures were generated by Rosetta. (TIF) [file pone.0205214.s011.tif]

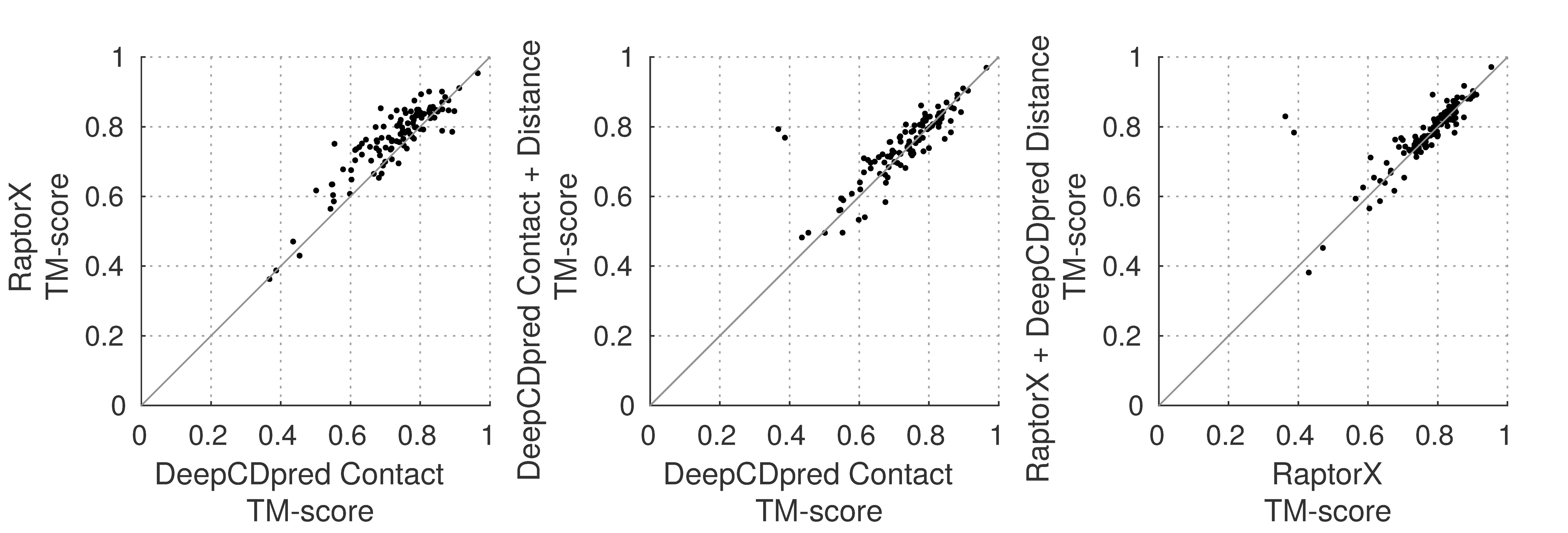

Supplement: S6 Fig — The calculations are for the test set of 108 proteins. The graphs show comparison of the TM-score with respect to experimental structures of the best models predicted using constraints from RaptorX, DeepCDpred contact only, DeepCDpred contact + distance and RaptorX contact + DeepCDpred distance predictions. For each test protein 100 structures were generated by Rosetta. (TIF) [file pone.0205214.s012.tif]

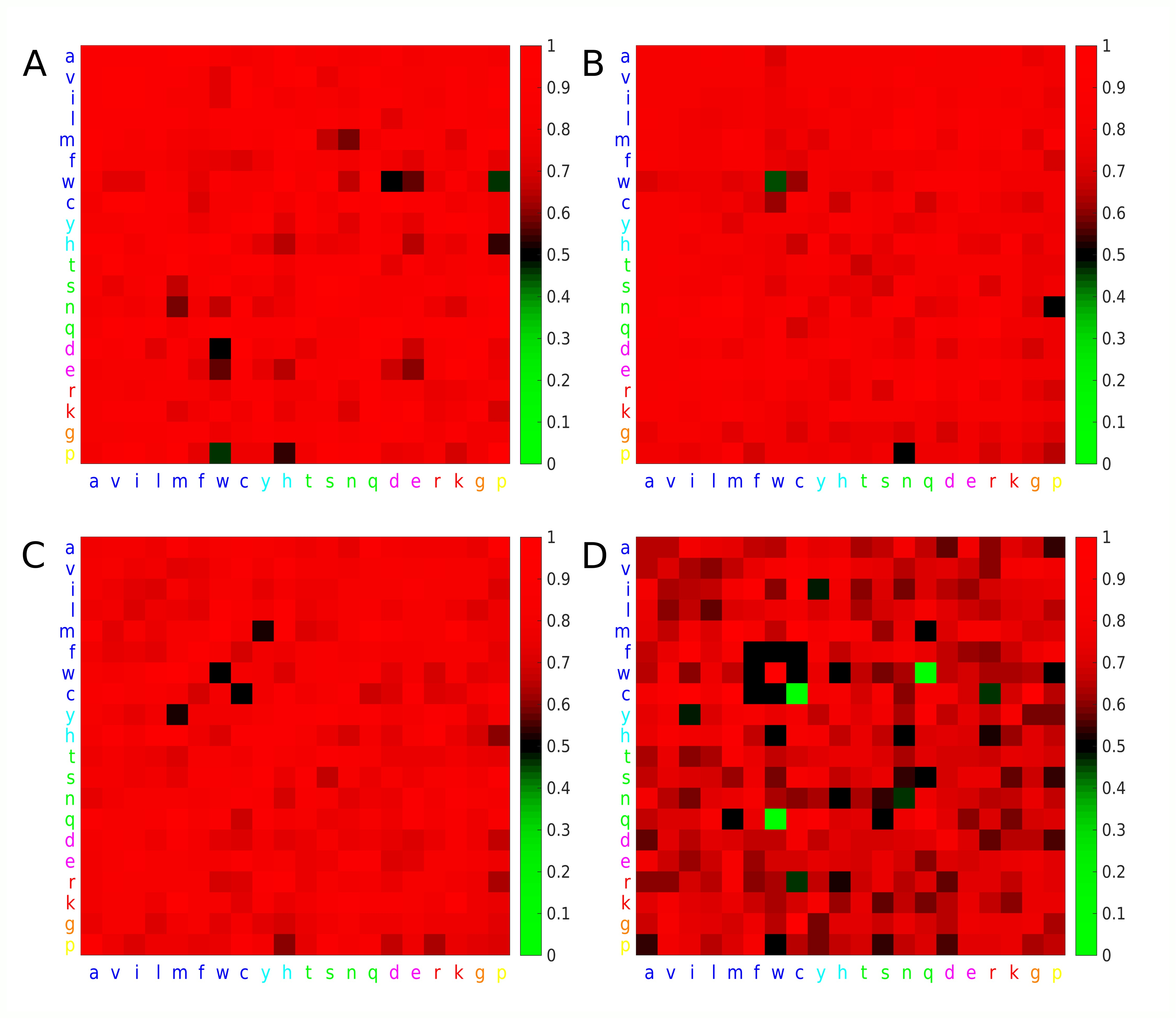

Supplement: S7 Fig — The scale is given on the right hand side for each plot. Precision is calculated as the number of correctly predicted contacts for that pair of amino acid types divided by the total number of contact predictions for that pair for the predictions with >= 0.7 network score. (TIF) [file pone.0205214.s013.tif]

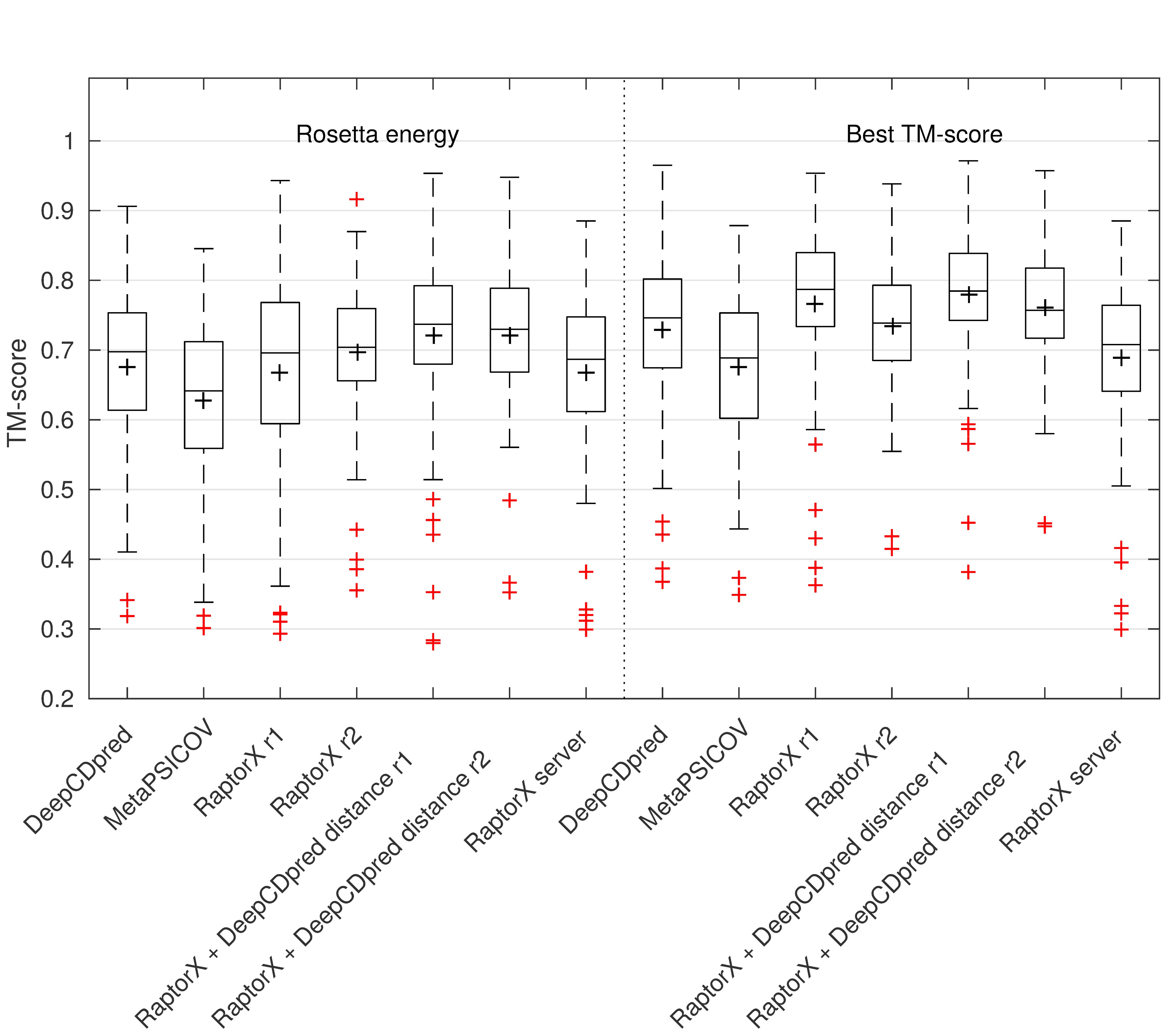

Supplement: S8 Fig — Structure predictions for Rosetta contact and Rosetta contact plus DeepCDpred distances were replicated (replica1 (r1) and replica2 (r2)). For Rosetta server predictions models were selected either by the lowest energy score (CNS score) or the best model among the 5 structures that the server provides. For all other prediction methods, models were selected either with the lowest Rosetta energy or the best TM-score. The calculations were performed for the test set of 108 proteins. The upper and the lower edges of the boxes indicate the 25th and 75th percentiles, respectively. The medians are shown with the central lines, the means are shown with black ‘+’ signs and the outliers are shown with red ‘+’ signs. Even though the first set of best models which were generated with the restraints of RaptorX contact predictions (RaptorX r1) are significantly better than the best models generated with DeepCDpred contact predictions, replication of the structure predictions with RaptorX contacts (RaptorX r2) resulted in no significantly different average TM-score than the predictions performed with DeepCDpred contacts (paired t-test p-value: 0.507). The results from the RaptorX server were on average worse than all other calculations except the use of MetaPSICOV contact restraints together with Rosetta, presumably because CNS, used by the RaptorX server, is not as good at modelling structures as Rosetta is. (TIF) [file pone.0205214.s014.tif]

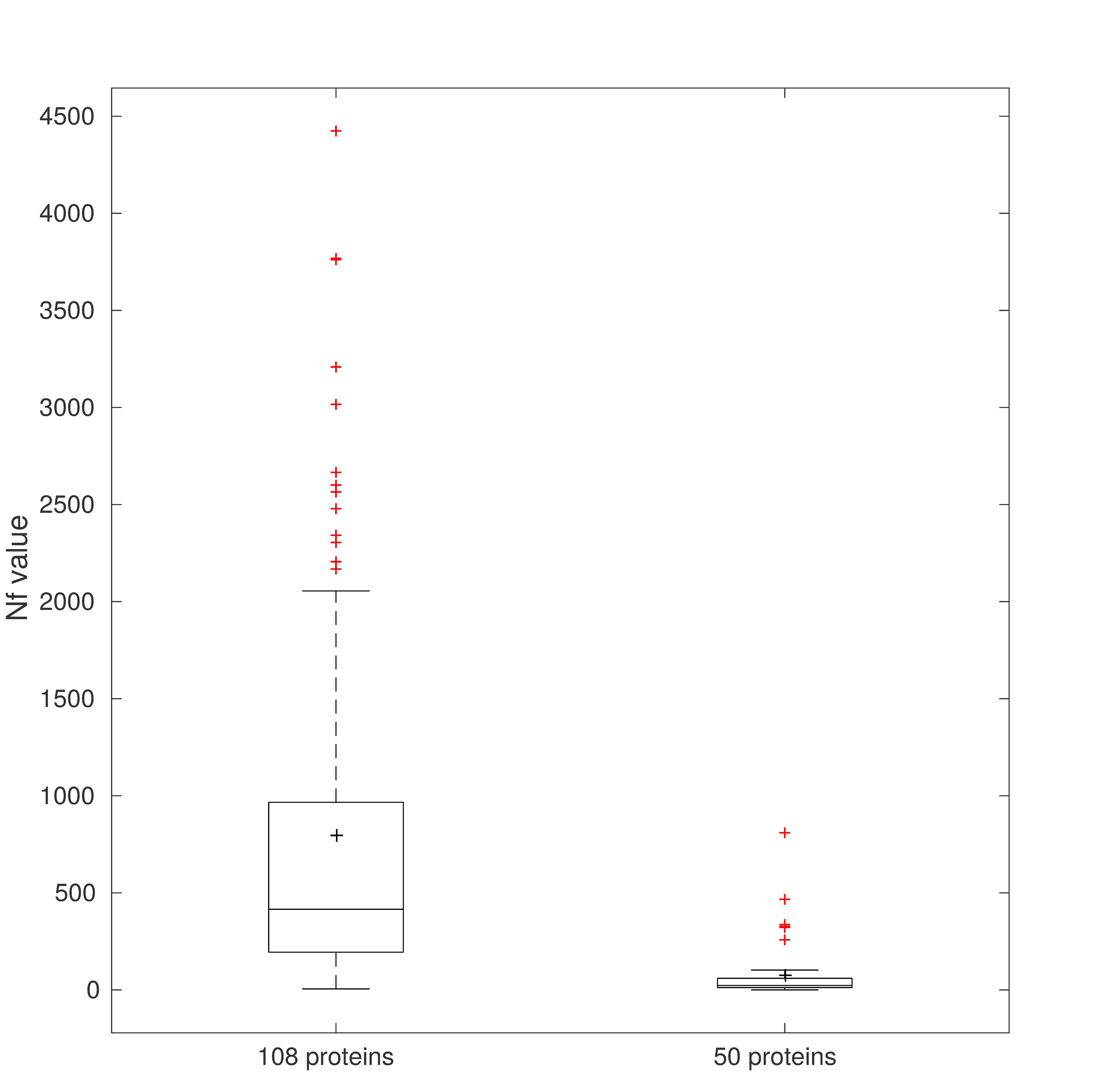

Supplement: S9 Fig — The upper and the lower edges of the boxes indicate the 25th and 75th percentiles, respectively. The medians are shown with the central lines, the means are shown with black ‘+’ signs and the outliers are shown with red ‘+’ signs. (TIF) [file pone.0205214.s015.tif]
